# Position-dependent carboxyl functionalization in covalent organic frameworks for selective photocatalytic CO_2_ reduction

**DOI:** 10.3389/fchem.2026.1892359

**Published:** 2026-06-22

**Authors:** Jiaxin Wang, Chunqiu Han, Liqun Ye

**Affiliations:** College of Materials and Chemical Engineering, Key Laboratory of Inorganic Nonmetallic Crystalline and Energy Conversion Materials China Three Gorges University, Yichang, China

**Keywords:** carboxyl-position isomerism, covalent organic frameworks, molecular structure regulation, photocatalytic CO_2_ reduction, product selectivity

## Abstract

Photocatalytic carbon dioxide (CO_2_) reduction offers a promising route for converting greenhouse gas into value-added solar fuels under mild conditions. However, the rational regulation of product selectivity, especially the directional conversion of CO_2_ toward either shallow reduction products such as carbon monoxide (CO) or deep reduction products such as methane (CH_4_), remains a major challenge. In this study, two carboxyl-position isomeric covalent organic frameworks covalent organic frameworks (COFs), TpBdda and TpBdad, were constructed to investigate how subtle differences in functional-group spatial arrangement affect CO_2_ photoreduction pathways. Although the two COFs possess similar framework compositions and overall morphologies, the different distribution of carboxyl groups leads to distinct structural features, pore environments, surface chemical properties, and photoinduced reaction behaviors. Photocatalytic CO_2_ reduction tests show that TpBdda mainly produces CO, with a CO evolution rate of approximately 5.2 μmol g^-1^ h^-1^ and nearly complete CO selectivity. In contrast, TpBdad exhibits a pronounced deep-reduction tendency, delivering CH_4_ as the dominant product with a CH_4_ evolution rate of approximately 1.8 μmol g^-1^ h^-1^ and a CH_4_ selectivity of about 90%. *In situ* diffuse reflectance infrared Fourier transform spectroscopy further reveals that TpBdda favors the formation and desorption of *CO-related intermediates, whereas TpBdad promotes the stabilization and subsequent hydrogenation of key intermediates, especially methoxy-related species, thereby facilitating CH_4_ formation. These results demonstrate that carboxyl-group spatial arrangement can effectively modulate intermediate evolution and product selectivity in COF-based photocatalytic CO_2_ reduction, providing a molecular-level strategy for designing selective organic photocatalysts.

## 1 Introduction

The excessive emission of CO_2_ has intensified global environmental and energy-related challenges, including climate change, carbon-cycle imbalance, and increasing pressure on sustainable energy systems. Converting CO_2_ into value-added fuels and chemicals is therefore considered an attractive strategy for simultaneously mitigating greenhouse gas emissions and realizing carbon resource utilization (Sathyanadh et al., 2026; Boubaker et al., 2025; Kumaravel et al., 2020; Vadivel et al., 2024; Dang et al., 2024; Vu et al., 2019). Among different CO_2_ conversion routes, photocatalytic CO_2_ reduction is particularly promising because it can directly utilize solar energy to drive chemical transformation under mild conditions, offering a sustainable route for solar-to-chemical energy conversion (Bellardita et al., 2021; Inoue et al., 1979; Tjandra et al.,. 2018).

However, efficient and selective photocatalytic CO_2_ reduction remains challenging because CO_2_ is a thermodynamically stable and kinetically inert molecule. Its conversion usually involves complex adsorption, activation, and multiple proton-coupled electron-transfer steps, which may lead to various products such as CO, HCOOH, CH_3_OH, and CH_4_ (Chang et al., 2016; Dang et al., 2024; He et al., 2024; Karamian and Sharifnia, 2016; Li et al., 2016; Xie et al., 2016). In particular, CO formation generally proceeds through a two-electron pathway, whereas CH_4_ generation requires a more demanding eight-electron process. Therefore, regulating the adsorption configuration, stabilization, and hydrogenation of key intermediates is essential for controlling product selectivity (Ji and Luo, 2016; Kong et al., 2020; Pang et al., 2026; Shkrob et al., 2012; Yang et al., 2011).

COFs have recently emerged as promising photocatalysts for CO_2_ reduction owing to their structural designability, ordered pore channels, high surface areas, and tunable electronic structures. Stable COF systems have demonstrated the feasibility of crystalline organic frameworks in solar-driven CO_2_ conversion (He et al., 2023; Sarkar et al., 2022; Yang et al., 2024). In addition, COFs can be constructed through dynamic covalent chemistry, allowing the rational design of pore environments, pi-conjugated skeletons, and functional groups (Segura et al., 2016; Shen et al.,. 2020; Song et al., 2022; Waller et al., 2015; Wang et al., 2025). These characteristics make COFs suitable platforms for investigating how molecular-level structural features influence CO_2_ adsorption, charge transfer, and intermediate evolution.

Previous studies have shown that the photocatalytic performance of COF-based or porous photocatalysts can be improved by optimizing charge separation, introducing active sites, modifying pore polarity, or regulating the local reaction environment (Chen et al., 2025; Guan et al., 2020; Liu et al., 2023; Yang et al., 2020). Functional groups are especially important because they can alter framework polarity, adsorption affinity, and the interaction between CO_2_-related intermediates and catalytic sites (An et al., 2019; Jiang et al., 2020; Kurisingal et al., 2022; Mohammed et al., 2020; Sharma et al., 2017). Nevertheless, most reported studies focus on enhancing overall activity, while the molecular origin of product selectivity, especially the switch between CO formation and deep reduction to CH_4_, remains insufficiently understood (Cheng et al., 2022; Li et al., 2020; Long et al., 2017; Ma et al., 2020; Prabhu et al., 2020).

Herein, two carboxyl-position isomeric COFs, TpBdda and TpBdad, were selected as model photocatalysts to clarify the effect of position-dependent carboxyl functionalization on photocatalytic CO_2_ reduction selectivity. Although these two COFs possess similar framework compositions, their different carboxyl distributions lead to distinct structural characteristics, surface chemical environments, and reaction behaviors. Photocatalytic tests reveal that TpBdda preferentially produces CO, whereas TpBdad exhibits a pronounced tendency toward CH_4_ formation. Combined with optical, photoelectrochemical, and *in situ* infrared analyses, this work demonstrates that carboxyl positional isomerism can modulate the formation, stabilization, and evolution of key intermediates, thereby directing CO_2_ photoreduction pathways toward either CO or CH_4_. This study provides molecular-level insight into the rational design of COF-based photocatalysts for selective CO_2_ conversion, complementing current efforts toward CH_4_-selective photocatalysis.

## 2 Materials and methods

### 2.1 Materials

2,4,6-Triformylphloroglucinol (Tp, AR 98%), 4,4′-diamino-[1,1′-biphenyl]-3,3′-dicarboxylic acid (AR 98%), and 3,3′-diamino-4,4′-dicarboxybiphenyl (AR 98%) were purchased from Shanghai Tengqian Chemical Reagent Co., Ltd. 1,4-Dioxane (AR 99%), mesitylene (AR 99%), and dichloromethane (AR 99%) were obtained from Macklin. Acetone (AR 99.5%) and hydrochloric acid (AR 99.5%) were purchased from Chengdu Kelong Chemical Reagent Co., Ltd. Glacial acetic acid (AR 99.5%) was purchased from Sinopharm Chemical Reagent Co., Ltd. All chemicals were used as received without further purification. Deionized water was used throughout the experiments.

### 2.2 Synthesis of TpBdda and TpBdad

TpBdda was synthesized through a solvothermal condensation reaction, following the general Schiff-base COF construction principle reported for imine-linked frameworks (Segura et al., 2016; Waller et al., 2015). Typically, 2,4,6-triformylphloroglucinol (Tp) and 4,4′-diamino-[1,1′-biphenyl]-3,3′-dicarboxylic acid were added into an ampoule at a molar ratio of 2:3. A mixed solvent of 1,4-dioxane and mesitylene with a volume ratio of 1:3 was then introduced, followed by ultrasonic dispersion to form a homogeneous suspension. Subsequently, 0.8 mL of 6 M acetic acid was rapidly added as the catalyst, and the mixture was further sonicated for 10 min. The ampoule was subjected to three freeze-pump-thaw cycles under liquid nitrogen, flame-sealed, and then heated at 120 °C for 3 days. After cooling to room temperature, the resulting solid was collected, thoroughly washed with acetone and dichloromethane, and dried under vacuum overnight.

TpBdad was prepared using a similar procedure, except that 3,3′-diamino-4,4′-dicarboxybiphenyl was used instead of 4,4′-diamino-[1,1′-biphenyl]-3,3′-dicarboxylic acid. The molar ratio of Tp to diamine monomer was also maintained at 2:3. The obtained product was washed with acetone and dichloromethane and dried under vacuum overnight. The two COFs were denoted as TpBdda and TpBdad according to the different spatial arrangements of carboxyl groups in the diamine monomers.

Note: the exact solvent system and reactor information should be checked against the original laboratory record before submission, because the thesis draft contains more than one description of the solvent system.

### 2.3 Photocatalytic CO_2_ reduction tests

The photocatalytic CO_2_ reduction performance of TpBdda and TpBdad was evaluated in a sealed quartz reactor. In a typical test, 10 mg of catalyst powder was dispersed in 10 mL of deionized water by ultrasonication to form a uniform suspension. Before irradiation, high-purity Ar was purged into the reactor for 5 min to remove residual air and oxygen. Then, 5 mL of CO_2_ was injected into the sealed reactor using a syringe. The reaction was conducted under irradiation from a 300 W Xe lamp without an optical filter for 2 h.

Gas samples were collected from the headspace of the reactor at 0.5 h intervals using a gas-tight syringe. For each sampling, 1 mL of gas was withdrawn and immediately injected into a gas chromatograph for product analysis. Each photocatalytic test was repeated two times, and the average values were used for comparison. Blank control experiments were performed under identical conditions in the absence of catalyst or CO_2_ to exclude possible contributions from residual carbon-containing impurities.

### 2.4 Product analysis

The gaseous products generated during photocatalytic CO_2_ reduction were analyzed by gas chromatography using a GC9790II gas chromatograph equipped with FID and TCD detectors with a molecular sieve 5A column, with Ar as the carrier gas. CO and CH_4_ were identified by comparing their retention times with those of standard gases. Quantitative analysis was performed using calibration curves established from high-purity CO and CH_4_ standard gases with a purity of 99.9%. The calibration curves were obtained under the same gas chromatographic conditions as those used for the photocatalytic tests. The production rates of CO and CH_4_ were calculated according to the detected gas amount, catalyst mass, and reaction time.

### 2.5 Photoelectrochemical measurements

Photoelectrochemical measurements were carried out on a CHI760E electrochemical workstation using a standard three-electrode configuration. The working electrode was prepared by mixing 10 mg of catalyst and 1 mg of ethyl cellulose with an appropriate amount of ethanol, followed by grinding to obtain a homogeneous slurry. The slurry was uniformly coated onto an FTO conductive glass substrate and dried at 120 °C for 2 h.

The catalyst-coated FTO glass, carbon rod, and Ag/AgCl electrode were used as the working electrode, counter electrode, and reference electrode, respectively. A 0.5 M Na_2_SO_4_ aqueous solution was used as the electrolyte. Electrochemical impedance spectroscopy (EIS) was performed at open-circuit potential or under a fixed bias over a frequency range of 10^5^–0.1 Hz. Transient photocurrent responses were recorded under intermittent light irradiation, with both light-on and light-off intervals set to 30 s.

### 2.6 Characterization

The crystalline structures of the samples were characterized by powder X-ray diffraction (XRD) using a SmartLab X-ray diffractometer at room temperature. The diffraction patterns were recorded over a 2θ range of 2°–40° at a scan rate of 8° min^−1^. Fourier-transform infrared spectroscopy (FTIR) was performed using a Infrared Fourier Spectrometer (Bruker, INVENIO-S) over the range of 4000–400 cm^−1^ to identify functional groups and framework bonding features. X-ray photoelectron spectroscopy (XPS) was conducted using an AXIS Supra spectrometer to analyze the surface elemental composition and chemical states of C, N, and O. Nitrogen adsorption-desorption measurements were performed at 77 K, and the specific surface areas were calculated by the Brunauer–Emmett–Teller method. The pore-size distributions were obtained by fitting the adsorption data using an appropriate model. Transmission electron microscopy (TEM) images were recorded on a JEM-2100F transmission electron microscope to observe the morphology and microstructure of the samples. Solid-state ^13^C nuclear magnetic resonance spectroscopy was used to investigate the chemical environments of carbon atoms in the COF frameworks. These combined characterization methods are commonly used to evaluate ordered porous organic frameworks and photocatalytic COF materials (Alahakoon et al., 2020; Wang et al., 2020).

*In situ* diffuse reflectance infrared Fourier transform spectroscopy was performed to monitor the surface intermediates during CO_2_ adsorption and photocatalytic CO_2_ reduction. The spectral evolution was used to identify CO_2_-related intermediates and to analyze the different reaction pathways over TpBdda and TpBdad.

## 3 Results and discussion

### 3.1 Structural and morphological characterization

**FIGURE 1:**
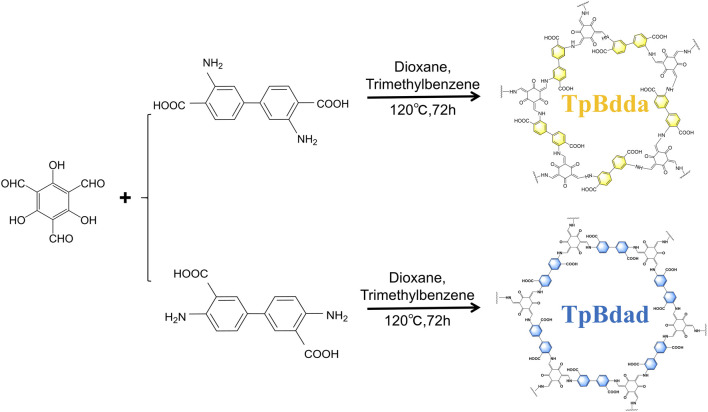
Synthetic route and structural design of TpBdda and TpBdad.

TpBdda and TpBdad were synthesized through solvothermal condensation between 2,4,6-triformylphloroglucinol (Tp) and two carboxyl-position isomeric diamine monomers, as schematically illustrated in Figure 1. The two COFs possess similar framework compositions, but differ in the spatial arrangement of carboxyl groups on the biphenyl units. Therefore, they provide a suitable model system for investigating how subtle functional-group positional isomerism affects the framework structure, local chemical environment, and photocatalytic CO_2_ reduction behavior.

**FIGURE 2:**
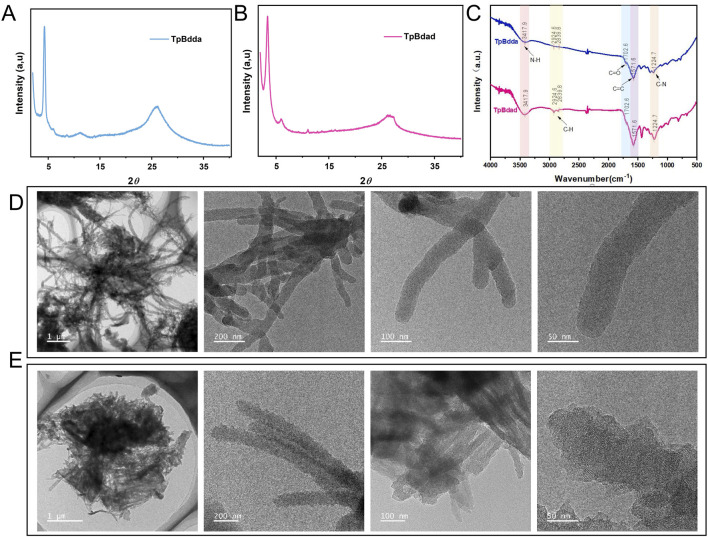
Structural and morphological characterization of TpBdda and TpBdad. XRD pattern of **(A)** TpBdda and **(B)** TpBdad. **(C)** FTIR spectra of TpBdda and TpBdad. **(D,E)** TEM images of TpBdda and TpBdad, representatively.

Powder X-ray diffraction (XRD) was first performed to examine the crystalline features of TpBdda and TpBdad. As shown in Figures 2A,B, both samples display distinct diffraction peaks in the low-angle region, indicating the formation of ordered COF frameworks. The main diffraction peak of TpBdad appears at approximately 2θ = 3.4°, whereas that of TpBdda is located at approximately 2θ = 4.2°. The shift of the main peak toward a lower angle for TpBdad suggests a larger characteristic lattice spacing, according to Bragg’s law. This result indicates that although the two COFs are constructed from similar building blocks, the different spatial positions of carboxyl groups significantly influence the framework packing mode and local ordered structure. In addition, both samples exhibit broad diffraction signals around 26°, which can be attributed to interlayer pi-pi stacking, a common feature in two-dimensional COF systems (Alahakoon et al., 2020; Dey et al., 2021; Segura et al., 2016).

Fourier-transform infrared spectroscopy (FTIR) was then used to confirm the formation of the organic framework and the retention of characteristic functional groups (Figure 2C). FTIR spectra of TpBdda and TpBdad exhibit similar main absorption peaks at approximately 1702.6 and 1571.6 cm^-1^, indicating that the two materials possess similar backbone structures. Meanwhile, slight differences in several characteristic bands suggest that carboxyl-position isomerism changes the local chemical environment of the framework. Such framework-level differences may influence the interaction between CO_2_ molecules, reaction intermediates, and active sites within the COF skeleton.

Transmission electron microscopy (TEM) was used to observe the morphology and microstructure of TpBdda and TpBdad (Figures 2D,E). The TEM images reveal that both samples exhibit aggregated organic framework morphologies, consistent with typical COF-based polymeric materials. The overall morphology of the two samples is relatively similar, suggesting that carboxyl-position isomerism does not completely change the macroscopic framework morphology.

**FIGURE 3:**
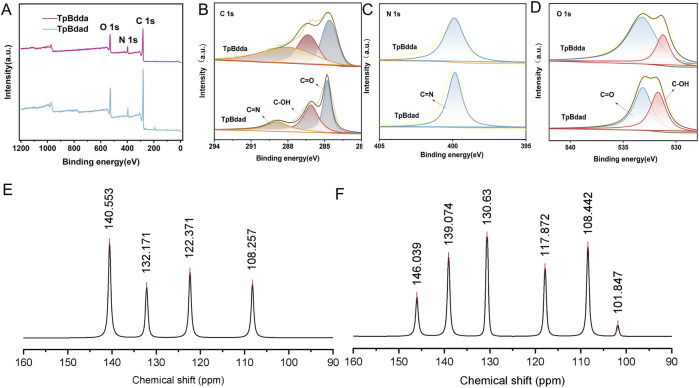
Surface chemical states and solid-state nuclear magnetic resonance analysis of TpBdda and TpBdad. High-resolution **(A)** XPS survey spectra, **(B)** C 1s, **(C)** N 1s, and **(D)** O 1s XPS spectra of TpBdda and TpBdad. Solid-state ^13^C NMR spectra of **(E)** TpBdda and **(F)** TpBdad.

X-ray photoelectron spectroscopy (XPS) was further conducted to analyze the surface elemental composition and chemical states of the two COFs (Figures 3A–D). The XPS survey spectra confirm the presence of C, N, and O elements in both TpBdda and TpBdad, without obvious impurity signals. This result verifies the successful construction of nitrogen- and oxygen-containing organic frameworks. The high-resolution C 1s spectrum in Figure 3B reveals that both TpBdda and TpBdad can be deconvoluted into multiple characteristic peaks, predominantly centered at ∼284.8, ∼286.0, and ∼288.0 eV. Specifically, the peak at lower binding energy is primarily assigned to conjugated C–C/C=C carbon in the framework backbones; the intermediate peak is associated with chemical environments such as C–N or C–OH; and the peak at higher binding energy corresponds to carbonyl-related carbon species. These results indicate that both samples possess a relatively intact organic conjugated framework and retain oxygen-containing functional groups. Further comparison demonstrates that TpBdda and TpBdad exhibit distinct differences in the relative intensities and peak shapes of the deconvoluted C 1s peaks, implying that the variation in carboxyl group positions modulates the electron cloud distribution around carbon atoms in the backbones, thereby leading to divergent surface carbon chemical environments.

The high-resolution N 1s spectrum in Figure 3C shows that both samples display a prominent characteristic peak at ∼400 eV, which can be attributed to C=N-related nitrogen species. This confirms that stable nitrogen-containing conjugated structures are formed in both TpBdda and TpBdad, verifying the successful construction of the target framework following the condensation reaction. While the N 1s peak positions of the two samples are generally comparable (indicating similar basic chemical environments for nitrogen), subtle differences in peak shapes suggest that the rearrangement of carboxyl groups can partially alter the local electronic environment around nitrogen sites. As shown in the high-resolution O 1s XPS spectra in Figure 3D, both samples can be deconvoluted into two characteristic peaks located at approximately 533.2 eV and 531.7 eV, which correspond to oxygen species in different chemical environments and are assigned to carbonyl oxygen and hydroxyl/phenolic hydroxyl-related oxygen species, respectively. This result confirms that abundant oxygen-containing functional groups are retained in both TpBdda and TpBdad. In comparison, TpBdad presents more distinct split O 1s peaks, revealing more remarkable differences in the local chemical environment of surface oxygen species.

Solid-state ^13^C nuclear magnetic resonance spectroscopy was employed to further identify the carbon chemical environments in TpBdda and TpBdad (Figures 3E,F). Both samples exhibit multiple carbon resonance signals in the high- and low-field regions, corresponding to different carbon species in the organic skeleton. The signal around 180 ppm can be assigned to carbonyl-related carbon species, while the signal near 170 ppm is associated with C=N carbon in the Schiff-base framework. The biphenyl moiety in TpBdda is ortho-substituted and possesses high symmetry, thus giving rise to aromatic carbon signals in the range of approximately 100–150 ppm. Within this interval, TpBdda displays four sets of aromatic carbon signals, which is two fewer than the number observed for the para-substituted TpBdad.

Both TpBdda and TpBdad exhibit distinct N_2_ adsorption-desorption isotherms, indicating the presence of pore structures in both materials. Notably, the two COFs differ significantly in pore size distribution: TpBdda shows a characteristic pore size centered at ∼2.3 nm, while TpBdad displays a dominant pore size at ∼3.9 nm (Supplementary Figure 1). The specific surface areas of TpBdda and TpBdad are determined to be 28.472 and 41.8646 m^2^ g^-1^ (Supplementary Table 1), respectively. These findings suggest that TpBdad, with its larger pore dimensions and higher specific surface area, not only facilitates CO_2_ molecule diffusion but also promotes the sustained adsorption of CO intermediates during CO_2_ reduction—favoring subsequent hydrogenation steps for CH_4_ formation.

Overall, the structural characterizations demonstrate that TpBdda and TpBdad were successfully synthesized as carboxyl-functionalized COF photocatalysts. More importantly, the subtle change in carboxyl-group spatial arrangement causes obvious differences in framework ordering, interlayer packing, and surface chemical environment. These structural differences provide an important basis for understanding the distinct photocatalytic CO_2_ reduction selectivity of TpBdda and TpBdad discussed below, as functionalized COF channels can influence both CO_2_ enrichment and intermediate evolution (An et al., 2019; Kurisingal et al., 2022; Sharma et al., 2017).

### 3.2 Photocatalytic CO_2_ reduction performance

The photocatalytic CO_2_ reduction performances of TpBdda and TpBdad were evaluated under identical reaction conditions to clarify the effect of carboxyl-group positional isomerism on catalytic activity and product selectivity. Before comparing the catalytic products, control experiments were carried out in the absence of catalyst or CO_2_. As summarized in Table 1, neither CO nor CH_4_ was detected under these control conditions, excluding the contribution of residual carbon-containing impurities or non-photocatalytic side reactions. In contrast, obvious carbon-containing reduction products were observed when the COF catalysts and CO_2_ were both present, confirming that CO and CH_4_ originated from photocatalytic CO_2_ conversion. Similar control experiments are essential for evaluating CO_2_ photoreduction because the reaction is sensitive to trace carbon sources and competing pathways (Habisreutinger et al., 2013; Li et al., 2016; Xie et al., 2016).

**TABLE 1:** Control experiments and product formation rates for photocatalytic CO_2_ reduction.

Reaction condition	CO rate (μmol g^-1^ h^-1^)	CH_4_ rate (μmol g^-1^ h^-1^)
Without catalyst	0	0
Without CO_2_	0	0
TpBdda + CO_2_	5.2	0
TpBdad + CO_2_	0.2	1.8

**FIGURE 4:**
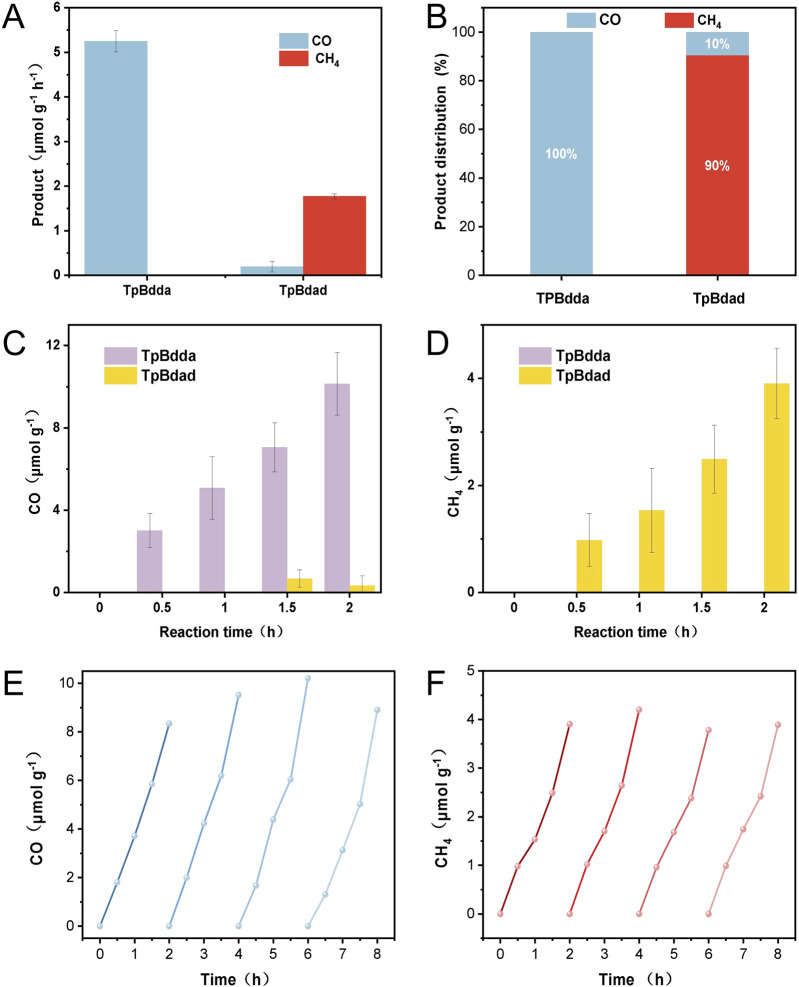
Photocatalytic CO_2_ reduction performance of TpBdda and TpBdad. **(A)** CO and CH_4_ evolution rates over TpBdda and TpBdad after 2 h irradiation. **(B)** Product selectivity difference between TpBdda and TpBdad in photocatalytic CO_2_ reduction. **(C)** Time-dependent CO production over TpBdda and TpBdad. **(D)** Time-dependent CH_4_ production over TpBdda and TpBdad. **(E)** Cycling stability test of TpBdda for photocatalytic CO_2_ reduction. **(F)** Cycling stability test of TpBdad for photocatalytic CO_2_ reduction.

The product formation rates after 2 h of irradiation are shown in Figure 4A. TpBdda exhibits CO as the dominant product, with a CO evolution rate of approximately 5.2 μmol g^-1^ h^-1^, while CH_4_ is barely detected. In sharp contrast, TpBdad mainly produces CH_4_, reaching a CH_4_ evolution rate of approximately 1.8 μmol g^-1^ h^-1^, accompanied by a small amount of CO with a formation rate of about 0.2 μmol g^-1^ h^-1^. The time-dependent product evolution profiles over TpBdda and TpBdad are further compared in Figures 4C,D. This obvious difference in product distribution indicates that the two COFs follow distinct CO_2_ photoreduction pathways despite their similar framework compositions.

The product selectivity further confirms the position-dependent catalytic behavior of the two carboxyl-functionalized COFs. As shown in Figure 4B, TpBdda shows nearly complete selectivity toward CO, whereas TpBdad exhibits a CH_4_ selectivity of approximately 90%, with CO accounting for only about 10% of the carbon-containing gaseous products. Since the formation of CO generally involves a two-electron reduction process, while CH_4_ generation requires a more demanding eight-electron/proton-coupled pathway, the preferential formation of CH_4_ over TpBdad suggests that this isomeric COF is more favorable for deep CO_2_ reduction (Cheng et al., 2022; Ji and Luo, 2016; Karamian and Sharifnia, 2016).

The time-dependent product evolution curves shown in Figures 4E,F further confirm the stable difference in product selectivity between TpBdda and TpBdad. For TpBdda, the CO amount continuously increases with irradiation time from 0.5 to 2.0 h, indicating that TpBdda can steadily promote CO formation during the reaction. For TpBdad, CH_4_ production also increases gradually with prolonged irradiation. More importantly, TpBdda maintains CO as the dominant product throughout the reaction, while TpBdad consistently gives CH_4_ as the major product. This result demonstrates that the selectivity difference is not caused by short-term fluctuation, but originates from the intrinsic structural difference induced by carboxyl-group positional isomerism.

Overall, these photocatalytic results clearly reveal that the spatial arrangement of carboxyl groups plays a decisive role in regulating the CO_2_ reduction pathway over COF photocatalysts. TpBdda tends to drive the reaction toward the shallow two-electron reduction product CO, whereas TpBdad facilitates further proton-coupled electron transfer and promotes deep reduction toward CH_4_. This position-dependent selectivity regulation is consistent with broader observations that local active-site environments, surface hydrophobicity, and intermediate stabilization can strongly influence CO_2_ photoreduction pathways (Li A. et al., 2019; Long et al., 2017; Ma et al., 2020; Wang et al., 2018).

### 3.3 Optical and photoelectrochemical properties

**FIGURE 5:**
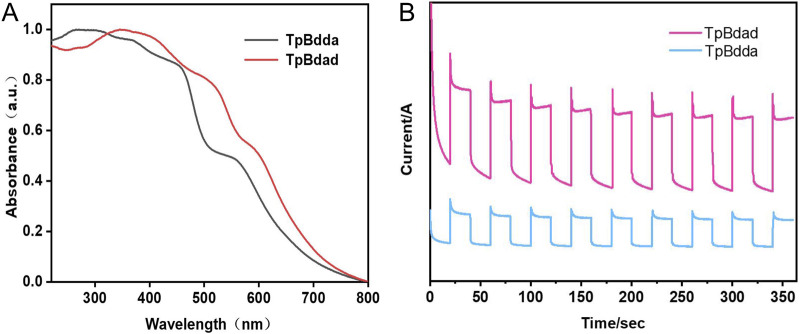
**(A)** UV-vis diffuse reflectance spectra of TpBdda and TpBdad. **(B)** Transient photocurrent responses of TpBdda and TpBdad under intermittent light irradiation.

To further elucidate the origin of the distinct behaviors of TpBdda and TpBdad during the photocatalytic CO_2_ reduction process, their optical absorption properties and charge carrier dynamics were first investigated *via* UV-Vis diffuse reflectance spectroscopy and transient photocurrent response measurements. As shown in Figure 5A, both TpBdda and TpBdad exhibit obvious absorption in the UV and visible-light regions, indicating that the two carboxyl-functionalized COFs possess the basic light-harvesting capability required for visible-light-driven photocatalysis. Compared with TpBdda, TpBdad shows a broader visible-light absorption range, with the absorption edge extending toward longer wavelengths. This red-shifted absorption suggests that the different spatial arrangement of carboxyl groups can modulate the electronic structure of the COF framework and improve visible-light utilization, which is a key factor for organic photocatalysts (Liu et al., 2023; Yang and Wang, 2018). Subsequently, the band structures of the two materials were further analyzed by combining Mott-Schottky plots and Tauc plots. As shown in Supplementary Figure 2, both TpBdda and TpBdad exhibit positive slopes in their Mott-Schottky curves, indicating that both materials possess n-type semiconductor characteristics. By extrapolating the curves to their intersection with the x-axis, the flat-band potentials of TpBdda and TpBdad are estimated to be approximately −0.29 V and −0.107 V (vs. NHE), respectively, revealing a distinct difference in their electron energy level positions. Meanwhile, the fitting results of the Tauc plots show that the optical band gaps of TpBdda and TpBdad are approximately 2.433 eV and 1.971 eV, respectively. These results demonstrate that the different arrangements of carboxyl groups in the framework not only affect the light absorption response of the materials but also further regulate their band structures and interfacial electron transfer behaviors.

The charge separation and migration behaviors of TpBdda and TpBdad were further evaluated by transient photocurrent response measurements (Figure 5B). Both COFs generate photocurrent signals under intermittent light irradiation, confirming that photoinduced electron-hole pairs can be produced and transported in the two frameworks. The difference in photocurrent intensity between TpBdda and TpBdad indicates that carboxyl-group positional isomerism affects the separation and migration efficiency of photogenerated carriers. In general, stronger photocurrent response and smaller interfacial resistance reflect more efficient separation and transfer of photoexcited charges, which can provide more available electrons for surface CO_2_ reduction reactions.

Collectively, optical and electrochemical studies demonstrate that the position of carboxyl groups in the COF backbones exerts a significant influence on light absorption and charge separation. These factors synergistically determine the overall performance of photocatalytic CO_2_ reduction. The key factors are more likely associated with the formation, stabilization, and subsequent hydrogenation of surface intermediates, which will be discussed in the following section based on *in situ* diffuse reflectance infrared Fourier transform spectroscopy (*in situ* DRIFTS) analysis.

### 3.4 Mechanistic insights into CO/CH_4_ selectivity

**FIGURE 6:**
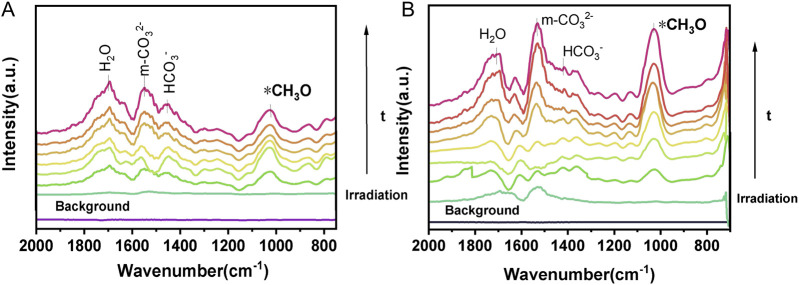
Mechanistic insights into position-dependent CO_2_ photoreduction selectivity over TpBdda and TpBdad. *In situ* DRIFTS spectra of **(A)** TpBdda and **(B)** TpBdad during photocatalytic CO_2_ reduction.

To clarify the origin of the distinct CO_2_ photoreduction selectivity over TpBdda and TpBdad, *in situ* DRIFTS was performed to monitor the evolution of surface intermediates during CO_2_ adsorption and photocatalytic reduction. As shown in Figures 6A,B, both TpBdda and TpBdad exhibit adsorption-related signals after CO_2_ introduction, indicating that the two carboxyl-functionalized COFs are capable of adsorbing and preliminarily activating CO_2_ molecules. Upon light irradiation, a series of new bands gradually appear and increase in the range of 2000–800 cm^-1^, suggesting the continuous formation and transformation of surface reaction intermediates during photocatalytic CO_2_ reduction.

Several characteristic bands can be observed during the reaction. The bands located at approximately 1612, 1543, 1425, and 1040 cm^−1^ can be associated with framework-related vibrations, monodentate carbonate species, bicarbonate species, and methoxy-related intermediates, respectively. In addition, the band around 1650 cm^−1^ is generally related to adsorbed water. These signals indicate that CO_2_ adsorption and activation on the COF surface involve carbonate/bicarbonate-type species and hydrogenated oxygen-containing intermediates, which are closely related to the subsequent reduction pathways (Anpo et al., 1995; Shkrob et al., 2012; Yang et al., 2011).

For TpBdda, distinct absorption bands gradually emerge at approximately 1690 and 1540 cm^−1^ under illumination, which can be assigned to *COOH and *CO-related intermediates, respectively. Meanwhile, a relatively weak band around 1040 cm^−1^ is also observed, suggesting the limited formation of hydrogenated oxygen-containing intermediates. Overall, the intermediate signals on TpBdda increase only moderately with irradiation time, indicating that the accumulation and further transformation of surface intermediates are relatively limited. This behavior suggests that CO_2_ reduction over TpBdda mainly follows a shallow reduction pathway from CO_2_ to *COOH and then to *CO, followed by rapid CO desorption. Therefore, the reaction over TpBdda tends to terminate at the two-electron CO formation stage, which agrees well with its nearly complete CO selectivity (Chang et al., 2016; Li et al., 2016; Xie et al., 2016).

In contrast, TpBdad shows more pronounced and continuously intensified DRIFTS signals during illumination, especially in the 1700–1500 cm^-1^ region and near 1040 cm^-1^. The stronger methoxy-related band over TpBdad indicates that this COF surface is more favorable for the formation and stabilization of hydrogenated intermediates associated with deep CO_2_ reduction. Compared with TpBdda, TpBdad enables a more continuous accumulation and transformation of key intermediates after CO_2_ adsorption, suggesting that the reaction intermediates have a longer residence time and a higher probability of undergoing further proton-coupled electron-transfer steps.

**FIGURE 7:**
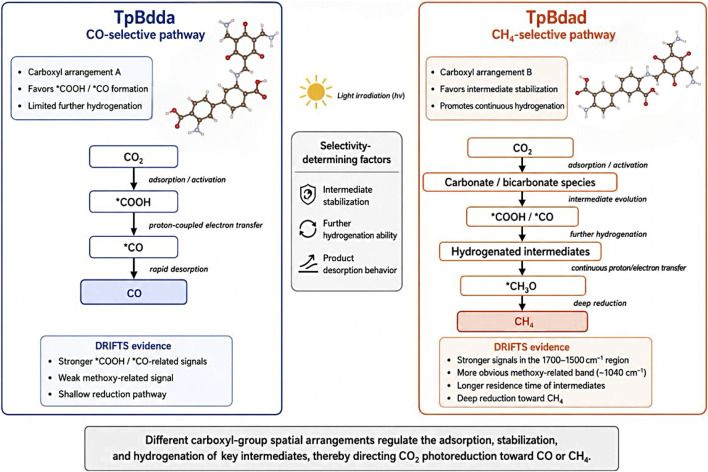
Investigation on the selectivity difference of TpBdda and TpBdad in photocatalytic CO_2_ reduction reaction.

Based on the above results, a possible position-dependent selectivity-regulation mechanism is proposed in Figure 7. In TpBdda, the spatial arrangement of carboxyl groups favors the formation of *COOH and *CO intermediates but does not effectively stabilize the intermediates required for further hydrogenation. As a result, *CO is more likely to desorb from the catalyst surface as CO, leading to high CO selectivity. In TpBdad, the different carboxyl-group arrangement modifies the local pore environment, surface chemical properties, and electronic structure, thereby enhancing the stabilization of reaction intermediates and promoting their further hydrogenation. The more obvious formation of methoxy-related species suggests that TpBdad can facilitate deeper reduction steps, eventually directing CO_2_ photoreduction toward CH_4_. The spatial arrangement of carboxyl groups enables the selective regulation of CO_2_ reduction products between CO and CH_4_ by modulating the adsorption stability of reaction intermediates and their subsequent hydrogenation activity.

Therefore, the different selectivity of TpBdda and TpBdad is not determined by a single factor such as light absorption or reduction potential. Instead, it results from the synergistic regulation of light harvesting, interfacial charge transfer, pore environment, and surface intermediate evolution induced by carboxyl positional isomerism. These findings demonstrate that the spatial arrangement of functional groups in COF frameworks plays a critical role in determining whether CO_2_ photoreduction stops at CO or proceeds toward CH_4_. This provides a molecular-level design strategy for selective COF-based photocatalysts and contributes to ongoing efforts to develop structurally defined organic materials for solar fuel production (He et al., 2023; Sarkar et al., 2022; Yeo et al., 2024).

## 4 Conclusion

In this work, two carboxyl-position isomeric covalent organic frameworks, TpBdda and TpBdad, were constructed as model photocatalysts to investigate the effect of position-dependent carboxyl functionalization on photocatalytic CO_2_ reduction selectivity. Although the two COFs possess similar framework compositions and overall morphologies, their different carboxyl-group spatial arrangements lead to distinct crystallinity, pore environments, surface chemical properties, optical responses, and charge-transport behaviors. Photocatalytic CO_2_ reduction tests reveal that TpBdda mainly promotes CO formation, with a CO evolution rate of approximately 5.2 μmol g^-1^ h^-1^ and nearly complete CO selectivity, whereas TpBdad favors deep CO_2_ reduction toward CH_4_, achieving a CH_4_ evolution rate of approximately 1.8 μmol g^-1^ h^-1^ and a CH_4_ selectivity of about 90%. Control experiments confirm that the detected CO and CH_4_ products originate from photocatalytic CO_2_ conversion rather than residual carbon-containing impurities. *In situ* DRIFTS analysis further indicates that TpBdda tends to facilitate the formation and rapid desorption of *CO-related intermediates, while TpBdad is more favorable for stabilizing key intermediates and promoting their continuous hydrogenation, thereby enabling CH_4_ formation. The methoxy (key intermediate for methane formation) peak of TpBdda is negligible, and the CO intermediate undergoes facile desorption, causing the reaction to terminate at the CO stage. In contrast, as illumination time extends, the characteristic methoxy peak of TpBdad intensifies synchronously and significantly. This allows TpBdad to stably trap intermediates and facilitate continuous multi-step hydrogenation, ultimately achieving deep reduction to CH_4_ through an eight-electron reduction pathway. These results demonstrate that the spatial arrangement of carboxyl groups in COF frameworks can effectively regulate intermediate evolution and product distribution during photocatalytic CO_2_ reduction. This study provides molecular-level insight into the rational design of COF-based photocatalysts and highlights position-dependent functional-group engineering as an effective strategy for selective solar-driven CO_2_ conversion.

## Data Availability

The original contributions presented in the study are included in the article/Supplementary Material, further inquiries can be directed to the corresponding authors.

## References

[B1] Alahakoon S. B. Diwakara S. D. Thompson C. M. Smaldone R. A. (2020). Supramolecular design in 2D covalent organic frameworks. Chem. Soc. Rev. 49, 1344–1356. 10.1039/c9cs00884e 32073066

[B2] An S. Xu T. Peng C. Hu J. Liu H. (2019). Rational design of functionalized covalent organic frameworks and their performance towards CO_2_ capture. RSC Adv. 9, 21438–21443. 10.1039/c9ra03487k 35521300 PMC9066184

[B3] Anpo M. Yamashita H. Ichihashi Y. Ehara S. (1995). Photocatalytic reduction of CO_2_ with H_2_O on various titanium oxide catalysts. J. Electroanal. Chem. 396, 21–26. 10.1016/0022-0728(95)04141-a

[B4] Bellardita M. Loddo V. Parrino F. Palmisano L. (2021). (Photo) electrocatalytic *versus* heterogeneous photocatalytic carbon dioxide reduction. ChemPhotoChem 5, 767–791. 10.1002/cptc.202100167

[B5] Boubaker S. Liu Z. Y. Mu Y. H. Zhan Y. S. (2025). Carbon dioxide emissions and environmental risks: long term and short term. Risk Anal. 45, 523–543. 10.1111/risa.14281 38375773

[B6] Chang X. Wang T. Gong J. (2016). CO_2_ photo-reduction: insights into CO_2_ activation and reaction on surfaces of photocatalysts. Energy & Environ. Sci. 9, 2177–2196. 10.1039/c6ee00383d

[B7] Chen X. Y. Liu C. Y. Yang T. Y. Jiang X. X. Yang Y. F. Su J. C. (2025). Cu atom pairs within covalent organic frameworks facilitate the photocatalytic reduction of CO_2_ to C_2_H_6_ . Appl. Catal. B-Environment Energy 377, 125499. 10.1016/j.apcatb.2025.125499

[B8] Cheng S. W. Sun Z. H. Lim K. H. Wibowo A. A. Zhang T. Du T. (2022). Emerging strategies for CO_2_ photoreduction to CH_4_: from experimental to data-driven design. Adv. Energy Mater. 12, 2200389. 10.1002/aenm.202200389

[B9] Dang H. Guan B. Chen J. Ma Z. Chen Y. Zhang J. (2024). Research status, challenges, and future prospects of carbon dioxide reduction technology. Energy & Fuels 38, 4836–4880. 10.1021/acs.energyfuels.3c04591

[B10] Dey K. Mohata S. Banerjee R. (2021). Covalent organic frameworks and supramolecular nano-synthesis. ACS Nano 15, 12723–12740. 10.1021/acsnano.1c05194

[B11] Guan X. Chen F. Fang Q. Qiu S. (2020). Design and applications of three dimensional covalent organic frameworks. Chem. Soc. Rev. 49, 1357–1384. 10.1039/c9cs00911f 32067000

[B12] Habisreutinger S. N. Schmidt-Mende L. Stolarczyk J. K. (2013). Photocatalytic reduction of CO_2_ on TiO_2_ and other semiconductors. Angew. Chem. Int. Ed. 52, 7372–7408. 10.1002/anie.201207199 23765842

[B13] He Z. Goulas J. Parker E. Sun Y. Zhou X.-D. Fei L. (2023). Review on covalent organic frameworks and derivatives for electrochemical and photocatalytic CO_2_ reduction. Catal. Today 409, 103–118. 10.1016/j.cattod.2022.04.021

[B14] He Y. M. Müller F. H. Palkovits R. Zeng F. Mebrahtu C. (2024). Tandem catalysis for CO_2_ conversion to higher alcohols: a review. Appl. Catal. B Environ. Energy 345, 123663. 10.1016/j.apcatb.2023.123663

[B15] Inoue T. Fujishima A. Konishi S. Honda K. (1979). Photoelectrocatalytic reduction of carbon dioxide in aqueous suspensions of semiconductor powders. Nature 277, 637–638. 10.1038/277637a0

[B16] Ji Y. Luo Y. (2016). Theoretical study on the mechanism of photoreduction of CO_2_ to CH_4_ on the anatase TiO_2_(101) surface. ACS Catal. 6, 2018–2025. 10.1021/acscatal.5b02694

[B17] Jiang X. Nie X. Guo X. Song C. Chen J. G. (2020). Recent advances in carbon dioxide hydrogenation to methanol *via* heterogeneous catalysis. Chem. Rev. 120, 7984–8034. 10.1021/acs.chemrev.9b00723 32049507

[B18] Karamian E. Sharifnia S. (2016). On the general mechanism of photocatalytic reduction of CO_2_ . J. CO_2_ Util. 16, 194–203. 10.1016/j.jcou.2016.07.004

[B19] Kong T. Jiang Y. Xiong Y. (2020). Photocatalytic CO_2_ conversion: what can we learn from conventional CO_x_ hydrogenation. Chem. Soc. Rev. 49, 6579–6591. 10.1039/c9cs00920e 32789318

[B20] Kumaravel V. Bartlett J. Pillai S. C. (2020). Photoelectrochemical conversion of carbon dioxide (CO_2_) into fuels and value-added products. ACS Energy Lett. 5, 486–519. 10.1021/acsenergylett.9b02585

[B21] Kurisingal J. F. Kim H. Choe J. H. Hong C. S. (2022). Covalent organic framework-based catalysts for efficient CO_2_ utilization reactions. Coord. Chem. Rev. 473, 214835. 10.1016/j.ccr.2022.214835

[B22] Li K. Peng B. Peng T. (2016). Recent advances in heterogeneous photocatalytic CO_2_ conversion to solar fuels. ACS Catal. 6, 7485–7527. 10.1021/acscatal.6b02089

[B23] Li A. Cao Q. Zhou G. Schmidt B. V. K. J. Zhu W. Yuan X. (2019a). Three-phase photocatalysis for the enhanced selectivity and activity of CO_2_ reduction on a hydrophobic surface. Angew. Chem. Int. Ed. 58, 14549–14555. 10.1002/anie.201908058 31418998 PMC7687246

[B24] Li X. D. Sun Y. F. Xu J. Q. Shao Y. Wu J. Xu X. (2019b). Selective visible-light-driven photocatalytic CO_2_ reduction to CH4 mediated by atomically thin CuIn_5_S_8_ layers. Nat. Energy 4, 690–699. 10.1038/s41560-019-0431-1

[B25] Li X. Yadav P. Loh K. P. (2020). Function-oriented synthesis of two-dimensional covalent organic frameworks: from 3D solids to 2D sheets. Chem. Soc. Rev. 49, 4835–4866. 10.1039/d0cs00236d 32490450

[B26] Liu S. Wang M. He Y. Cheng Q. Qian T. Yan C. (2023). Covalent organic frameworks towards photocatalytic applications: design principles, achievements, and opportunities. Coord. Chem. Rev. 475, 214882. 10.1016/j.ccr.2022.214882

[B27] Long R. Li Y. Liu Y. Chen S. Zheng X. Gao C. (2017). Isolation of Cu atoms in Pd lattice: forming highly selective sites for photocatalytic conversion of CO_2_ to CH_4_ . J. Am. Chem. Soc. 139, 4486–4492. 10.1021/jacs.7b00452 28276680

[B28] Ma Y. J. Tang Q. Sun W. Y. Yao Z. Zhu W. Li T. (2020). Assembling ultrafine TiO2 nanoparticles on UiO-66 octahedrons to promote selective photocatalytic conversion of CO_2_ to CH4 at a low concentration. Appl. Catal. B Environ. 270, 118856. 10.1016/j.apcatb.2020.118856

[B29] Mohammed A. K. Usgaonkar S. Kanheerampockil F. Karak S. Halder A. Tharkar M. (2020). Connecting microscopic structures, mesoscale assemblies, and macroscopic architectures in 3D-printed hierarchical porous covalent organic framework foams. J. Am. Chem. Soc. 142, 8252–8261. 10.1021/jacs.0c00555 32279483

[B30] Pang Z. Y. Wang B. Di J. Li Y. J. She Y. B. Li H. M. (2026). Photocatalytic carbon dioxide reduction to value-added hydrocarbons products. Coord. Chem. Rev. 557, 217705. 10.1016/j.ccr.2026.217705

[B31] Prabhu P. Jose V. Lee J.-M. (2020). Heterostructured catalysts for electrocatalytic and photocatalytic carbon dioxide reduction. Adv. Funct. Mater. 30, 1910768. 10.1002/adfm.201910768

[B32] Sarkar P. Chowdhury I. H. Das S. Islam S. M. (2022). Recent trends in covalent organic frameworks (COFs) for carbon dioxide reduction. Mater. Adv. 3, 8063–8080. 10.1039/d2ma00600f

[B33] Sathyanadh A. Esfandiari H. Bourgeois T. Schwinger J. Bergman T. Partanen A. I. (2026). Efficacy of individual and combined terrestrial and marine carbon dioxide removal. Environ. Res. Lett. 21, 014032. 10.1088/1748-9326/ae2af5

[B34] Segura J. L. Mancheno M. J. Zamora F. (2016). Covalent organic frameworks based on schiff-base chemistry: synthesis, properties and potential applications. Chem. Soc. Rev. 45, 5635–5671. 10.1039/c5cs00878f 27341661

[B35] Sharma A. Malani A. Medhekar N. V. Babarao R. (2017). CO_2_ adsorption and separation in covalent organic frameworks with interlayer slipping. CrystEngComm 19, 6950–6963. 10.1039/c7ce01647f

[B36] Shen H. Peppel T. Strunk J. Sun Z. (2020). Photocatalytic reduction of CO_2_ by metal-free-based materials: recent advances and future perspective. Sol. RRL 4, 1900546. 10.1002/solr.201900546

[B37] Shkrob I. A. Marin T. W. He H. Zapol P. (2012). Photoredox reactions and the catalytic cycle for carbon dioxide fixation and methanogenesis on metal oxides. J. Phys. Chem. C 116, 9450–9460. 10.1021/jp300122v

[B38] Song K. S. Fritz P. W. Coskun A. (2022). Porous organic polymers for CO_2_ capture, separation and conversion. Chem. Soc. Rev. 51, 9831–9852. 10.1039/d2cs00727d 36374129 PMC9703447

[B39] Subrahmanyam M. Kaneco S. Alonso-Vante N. (1999). A screening for the photoreduction of carbon dioxide supported on metal oxide catalysts for C_1_-C_3_ selectivity. Appl. Catal. B Environ. 23, 169–174. 10.1016/s0926-3373(99)00079-x

[B40] Tjandra A. D. Huang J. (2018). Photocatalytic carbon dioxide reduction by photocatalyst innovation. Chin. Chem. Lett. 29, 734–746. 10.1016/j.cclet.2018.03.017

[B41] Vadivel D. Dondi D. Capodaglio A. G. (2024). Present achievements and future directions of advanced carbon dioxide reduction strategies. Curr. Opin. Chem. Eng. 45, 101029. 10.1016/j.coche.2024.101029

[B42] Vu N. N. Kaliaguine S. Do T. O. (2019). Critical aspects and recent advances in structural engineering of photocatalysts for sunlight-driven photocatalytic reduction of CO_2_ into fuels. Adv. Funct. Mater. 29, 1901825. 10.1002/adfm.201901825

[B43] Waller P. J. Gandara F. Yaghi O. M. (2015). Chemistry of covalent organic frameworks. Accounts Chem. Res. 48, 3053–3063. 10.1021/acs.accounts.5b00369 26580002

[B44] Wang K. F. Zhang L. Su Y. Shao D. Zeng S. Wang W. (2018). Photoreduction of carbon dioxide of atmospheric concentration to methane with water over CoAl-layered double hydroxide nanosheets. J. of Mater. Chem. A 6, 8366–8373. 10.1039/c8ta01309h

[B45] Wang Z. Zhang S. Chen Y. Zhang Z. Ma S. (2020). Covalent organic frameworks for separation applications. Chem. Soc. Rev. 49, 708–735. 10.1039/c9cs00827f 31993598

[B46] Wang Q. Yuan W. J. Zhan Y. H. Chi X. Y. Qi D. P. Zhang X. L. (2025). Tailoring the structural design of covalent organic frameworks for enhanced photocatalytic carbon dioxide reduction: a review. Renew. & Sustain. Energy Rev. 217, 115754. 10.1016/j.rser.2025.115754

[B47] Xie S. Zhang Q. Liu G. Wang Y. (2016). Photocatalytic and photoelectrocatalytic reduction of CO_2_ using heterogeneous catalysts with controlled nanostructures. Chem. Commun. 52, 35–59. 10.1039/c5cc07613g 26540265

[B48] Yang X. Wang D. (2018). Photocatalysis: from fundamental principles to materials and applications. ACS Appl. Energy Mater. 1, 6657–6693. 10.1021/acsaem.8b01345

[B49] Yang C.-C. Vernimmen J. Meynen V. Cool P. Mul G. (2011). Mechanistic study of hydrocarbon formation in photocatalytic CO_2_ reduction over Ti-SBA-15. J. Catal. 284, 1–8. 10.1016/j.jcat.2011.08.005

[B50] Yang Q. Luo M. Liu K. Cao H. Yan H. (2020). Covalent organic frameworks for photocatalytic applications. Appl. Catal. B Environ. 276, 119174. 10.1016/j.apcatb.2020.119174

[B51] Yang J. L. Chen Z. H. Zhang L. Zhang Q. C. (2024). Covalent organic frameworks for photocatalytic reduction of carbon dioxide: a review. ACS Nano 33, 21804–21835. 10.1021/acsnano.4c06783 39116003

[B52] Yeo C. I. Tan Y. S. Awan H. T. A. Hanan A. Wong W. P. Walvekar R. (2024). A review on the advancements in covalent organic frameworks for photocatalytic reduction of carbon dioxide. Coord. Chem. Rev. 521, 216167. 10.1016/j.ccr.2024.216167

