# Supplementary material for: Position-dependent carboxyl functionalization in covalent organic frameworks for selective photocatalytic CO2 reduction
Source: Front Chem. 2026 Jun 22;14:1892359. doi: 10.3389/fchem.2026.1892359 (PMC13333696; doi:10.3389/fchem.2026.1892359)
Supplement: Supplementary file 1 [file DataSheet1.docx]

Supplementary Material

# Supplementary Figures

## Pore structure characterization


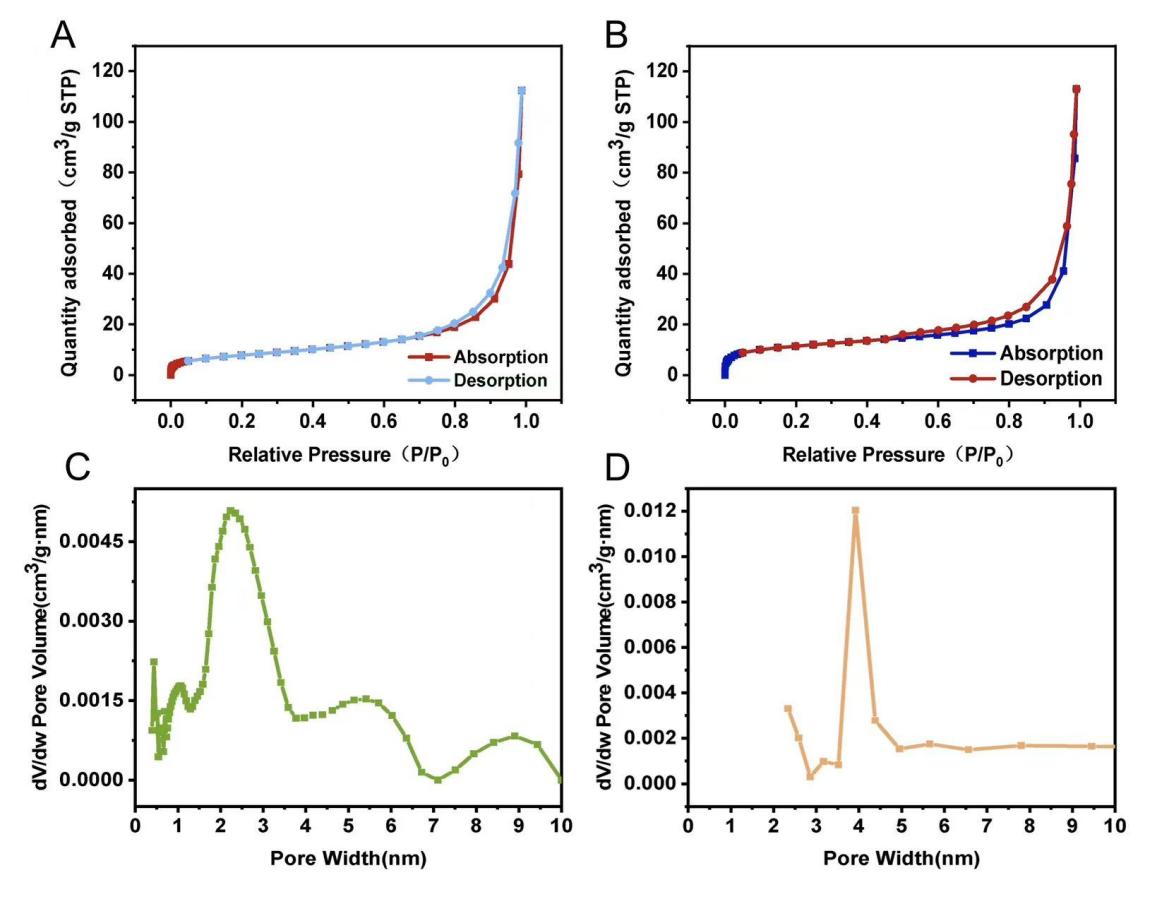


**Supplementary Figure 1.** (A) N_2_ adsorption-desorption isotherm of TpBdda; (B) N_2_ adsorption-desorption isotherm of TpBdad; (C) Pore size distribution profile of TpBdda; (D) Pore size distribution profile of TpBdad

## Band structure characterization of TpBdda and TpBdad


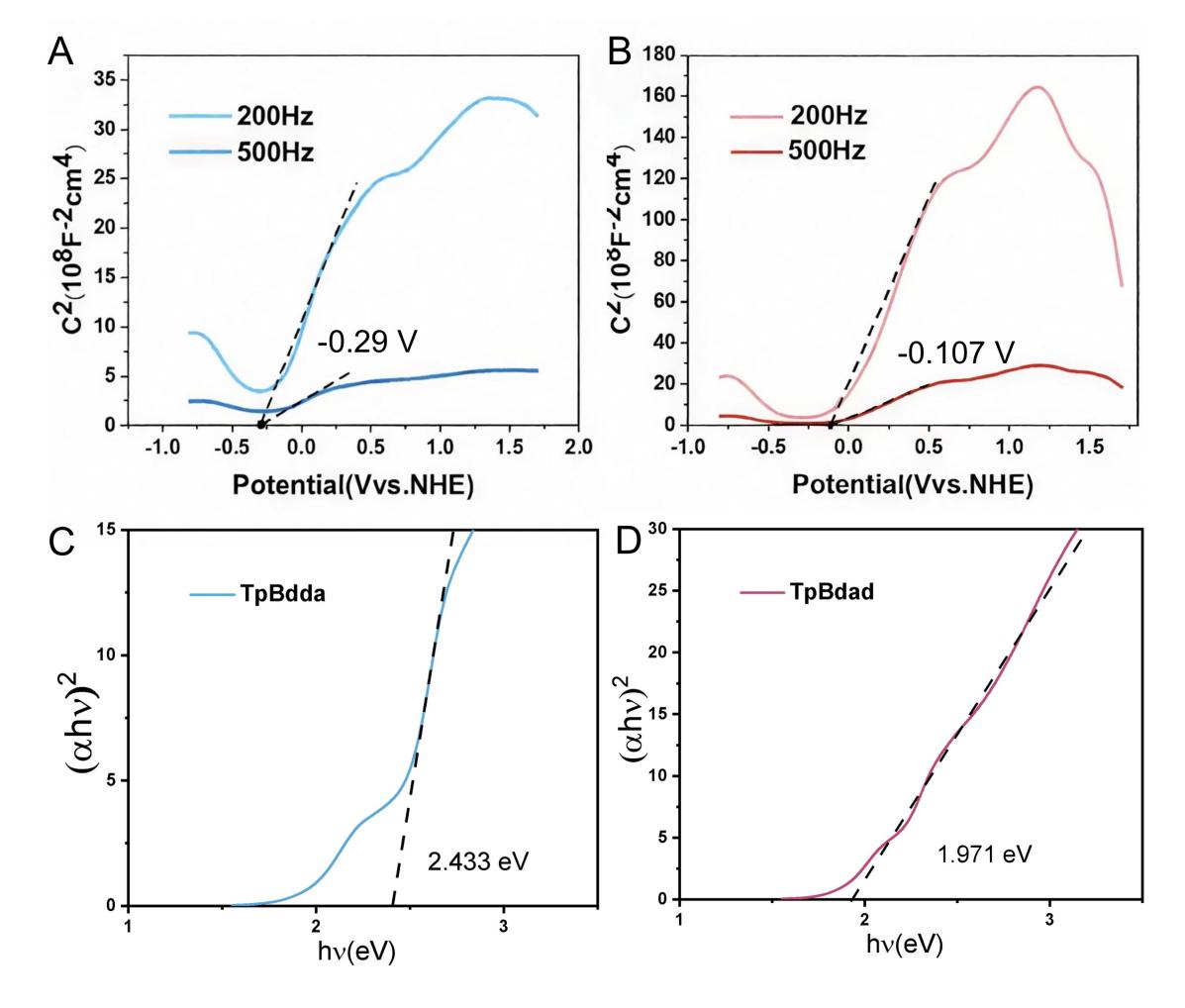


**Supplementary Figure 2.** (A) Mott–Schottky plots of TpBdda at varying frequencies; (B) Mott-Schottky plots of TpBdad at varying frequencies; (C) Tauc plot of TpBdda; (D) Tauc plot of TpBdad

# Supplementary Table

## Specific surface areas

**Supplementary Table 1** The specific surface areas of TpBdda and TpBdad.

| Samples | Specific surface area/m^2^ g^-1^） |
| --- | --- |
| TpBdda | 28.472 |
| TpBdad | 41.8646 |
